# Neural Assemblies as Precursors for Brain Function

**DOI:** 10.3390/neurosci3040046

**Published:** 2022-11-10

**Authors:** Kieran Greer

**Affiliations:** Distributed Computing Systems, Belfast BT1 9JY, UK; kgreer@distributedcomputingsystems.co.uk

**Keywords:** neural, brain, structural intelligence, cell expression, evolution

## Abstract

This concept paper gives a narrative about intelligence from insects to the human brain, showing where evolution may have been influenced by the structures in these simpler organisms. The ideas also come from the author’s own cognitive model, where a number of algorithms have been developed over time and the precursor structures should be codable to some level. Through developing and trying to implement the design, ideas like separating the data from the function have become architecturally appropriate and there have been several opportunities to make the system more orthogonal. Similarly for the human brain, neural structures may work in-sync with the neural functions, or may be slightly separate from them. Each section discusses one of the neural assemblies with a potential functional result, that cover ideas such as timing or scheduling, structural intelligence and neural binding. Another aspect of self-representation or expression is interesting and may help the brain to realise higher-level functionality based on these lower-level processes.

## 1. Introduction

This paper describes some neural representations that may be helpful for realising intelligence in the human brain. The ideas come from the author’s own cognitive model, where a number of algorithms have been developed over time. Through developing and trying to implement the design, ideas like separating the data from the function have become architecturally appropriate and there have been several opportunities to make the system more orthogonal. Similarly for the human brain, neural structures may work in-sync with the neural functions, or may be slightly separate from them. Having more than 1 information flow actually makes the problem of how the human brain works much easier to solve. Another aspect of self-representation or expression is interesting and may help the brain to realise higher-level functionality based on these lower-level processes, maybe even natural language itself. The cognitive model is still at the symbolic level and so the neural representations are also at this level. The neuron discussion is therefore at a statistical or biophysical level rather than a biological one.

The rest of the paper is organised as follows: Section 2 describes some related work. Then, the other sections discuss one of the neural assemblies with a potential functional result. Section 3 describes earlier work on a timer or scheduler. Section 4 describes how intelligence may be inherent in the neuron structure. Section 5 describes how the neural binding problem can be simplified. Section 6 describes some aspects of the author’s own cognitive model that have influenced the writing of this paper and Section 7 describes how natural language may have evolved naturally from similar structures. Finally, Section 8 gives some conclusions on the work.

## 2. Related Work

This paper is based mostly on the author’s own cognitive model, who comes from a computer science background. It has been described in detail, in particular, in the paper series ‘New Ideas for Brain Modelling’ 1–7 [1,2,3,4]. Most of the Artificial Intelligence technology is therefore described in the following sections, but a background to supporting biological work is described here. Supporting biological work includes [5,6,7,8] and also biophysical or statistical work, for example [9,10]. Having more than 1 information flow has been studied extensively. For example, the paper [8] describes that more than one type of sodium channel can be created and that they interact with each other, producing a variable signal. Small currents are involved, even for Ion channels and they work at different potentials, etc. It is also described how neurons can change states and start firing at different rates. Memory is a key topic, where the paper [6] describes that positive regulators can give rise to the growth of new synaptic connections and this can also form memories. There are also memory suppressors, to ensure that only salient features are learned. Long-term memory endures by virtue of the growth of new synaptic connections, which is a structural change. There is also some mathematical background, where the paper [7] was the basis for the simulation equation of [2] and the book [5] is a critical work on the neocortex and higher brain functions. The argument for this paper is still at the symbolic level, where the papers [9,10] both try to describe how the brain might organise itself through statistical processes.

The paper [11] may have developed a synaptic model, based on the themes of this paper. The authors state that recent neuroscience evidences indicate that astrocytes interact closely with neurons and participate in the regulation of synaptic neurotransmission, which has motivated new perspectives for the research of stigmergy in the brain. Additionally, that each astrocyte contains hundreds or thousands of branch microdomains, and each of them encloses a synapse, where distance between coupled branch microdomains is a critical factor. They also carry out tests to show the importance of regular distances between neurons.

The pioneering work of Santiago Ramón y Cajal (http://www.scholarpedia.org/article/Santiago_Ramón_y_Cajal (accessed on 18 October 2022)) may be supportive, in relation to pacemaker cells [12] and discrete units. Then, a new theory by Tsien [13] suggests that perineuronal nets, discovered by Golgi (https://en.wikipedia.org/wiki/Camillo_Golgi (accessed on 18 October 2022)) may be key to how the brain stores long-term memories and it is the basis for the cognitive model of this paper as well. The idea of an extracellular matrix was actually rejected by Cajal, where a discrete brain function was preferred. Neural binding is discussed in one section, but with a view to making it less holistic, where contrasting biological work might include [14,15]. Other biological work on simpler organisms includes [16,17,18,19,20,21,22,23] and is noted in the following sections.

## 3. Timing

**Figure 1:**
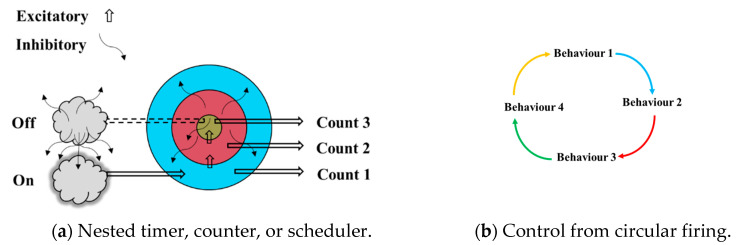
Nested Scheduling [2], or circular scheduling.

This was an early discovery for an automatic scheduler or counter [2]. It is not as relevant to the other sections, but it does offer an automatic construction for an intelligent process. The paper considered using nested structures, not only for concept ensembles, but also for more mechanical processes. If the structure fires inwards, then the rather obvious idea would be that an inner section would fire inhibitors outwards that would eventually switch off the source to its activation. It may also fire positively inwards, when the process would repeat with the next inner section, and so on. This switching on and off of nested sections could lead to a type of scheduling or timing, if each section also sent a signal somewhere else. This is illustrated in Figure 1, which also shows how a circular arrangement can behave in the same way [16]. A simulation of this process was run using Equation (1) that processed at a pattern level, not a synapse level and is a simplified version of an equation from [7]. It showed the expected result of how the pattern excitatory values would flow through the nested levels, rather like a colonic movement, for example. These tests therefore only considered the excitatory/inhibitory part, to measure how the patterns would switch on and off through their interactions.

The test equation, introduced in [2], is repeated next:

(1) $$X_{it}=\sum_{p=1}^{Pi}E_{p}t-(\sum_{k=Pj}^{l}\sum_{y=1}^{m}\sum_{j=1}^{n}\left(Hjy*\delta \right))$$

where *y* ≠ *t* and i∈Pi and not j∈Pi, and

X_*it*_ = total input signal for neuron *i* at time *t*.

*E_p_* = total excitatory input signal for neuron *p* in pattern *P*.

*H_jy_* = total inhibitory input signal for neuron *j* at time *y*.

*δ* = weights inhibitory signal.

*t* = time interval for firing neuron.

*y* = time interval for any other neuron.

*n* = total number of neurons.

*m* = total number of time intervals.

*l* = total number of active patterns.

*P_i_* = pattern for neuron *i*.

*P* = total number of patterns.

A schematic of the total signal input to each neuron over 3 time periods, is given in Table 1. To save space, repeating neuron values are not shown.

This is therefore one of the most basic processes in a human and other much simpler animals. The elegans worm is much studied, for example, because it has a brain of only about 300 neurons that can be mapped accurately. The paper [16] found that ‘most active neurons share information by engaging in coordinated, dynamical network activity that corresponds to the sequential assembly of motor commands’. While the neural assemblies might not be nested, there is a circular arrangement to the behavioural network [17] that produces a sequence of behaviours. It is likely that the nested arrangement would be more powerful, however. The worm also has pacemaker-like cells to activate some behaviours [18].

## 4. From Neuron to Network

It is proposed in this paper that the neuron and the brain network use a similar functionality that derives from the structure. The architecture for the neuron is the standard one of soma body, dendrites as the input channels and the axon as the output channel. The input is an amalgamation of other neuron signals, which gets sorted in the dendrites and soma into a more specific signal that is then transferred to the axon for sending to other neurons. In essence, the process converts signals from being set-based to being type-based. This would be a well-accepted filtering process and it is argued that the conversion from a set-based ‘scenario’ to more specific and local types in the output, is key to generating intelligence from the structure. This may also help to justify the author’s own ensemble-hierarchy structure ([4] and earlier papers). Note that a type however is simply something more singular. It does not have to represent only one input signal, for example, but represents a consistent set of input values. With this architecture therefore, the signal from one neuron to another must also be type-based, but the ensemble input is a set of signals from several neurons. Each set may get sorted differently and therefore create a different set of output types, and so the neuron can be part of more than 1 pattern at any time, where the timing of receiving a signal type would be important.

The neuron can therefore be part of several patterns, making it quite flexible with regard to the information flow. If the input has a chemical bias, for example, then that may allow the synapses related to that particular chemical type, to form and gather sufficient energy to release the signal to other neurons. This would be stigmergic [11,19] in nature. For example, if a neuron fires a signal of a particular type and that is then sent through a network and back to the neuron again, the neuron will already be able to reinforce its current state. It may also now be prepared for the signal [5] and be able to emit it more easily again. This means that the output from a neuron can be sent anywhere, but as with biophysics [10], where there are similar concentrations of a particular type, then the network will start to fire and form patterns relating to that type. Ants or Termites, for example, are able to share information locally, from the stigmergic build-up of chemical signals and this is also optimised for journey time [20]. Another paper [21] discovered that the ant can use different chemical types to indicate ‘road-signs’ inside of the nest and they use this to spatially segregate. They therefore recognise different types.

**Figure 2:**
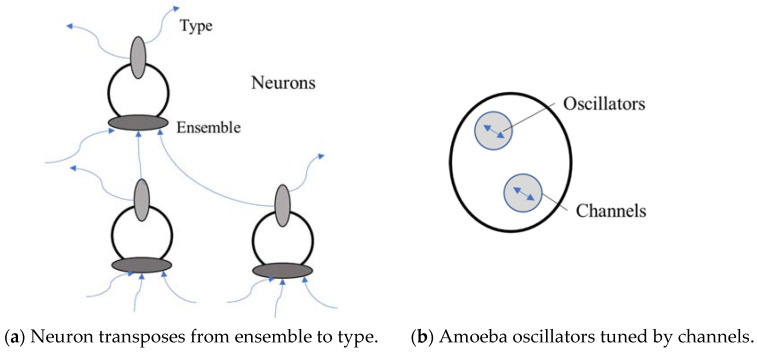
Network of neurons, with a comparative amoeba.

This architecture still does not require any intelligence. Thinking about simpler organisms again, at the SAI’14 conference (Prof. Chen’s Talk, SAI’14), the speaker asked ‘why’ an amoeba has a memory and not just how. If it is not to think, then it must be for a functional reason and this function must have evolved from the genetic makeup of the amoeba, hinting that such a mechanism can evolve naturally. So, why did the amoeba develop a memory? The obvious answer would be an evolutionary development for survival, but the author would like to postulate further and guess that it may also be because a living organism has a need to express itself. This desire may go back to the reproduction process itself. An earlier paper argued that true AI cannot be realised because we cannot simulate the living aspect of human cells [24], for example, and that may include this expressive nature. As with a stigmergic build-up, if the amoeba has set itself-up for a particular type of input, maybe it does not react to other input immediately but can only react to the specific input again, even after a short delay. The paper [23] models the amoeba behaviour as a memristor, which is a similar type of electronic circuit. They note that: the model however does not fully explain the memory response of the amoeba and does not take into account the fact that, at a microscopic level, changes in the physiology of the organism occur independently of the biological oscillators. These changes also occur over a finite period of time and must be dependent on the state of the system at previous times. This last point is particularly important: it is in fact this state-dependent feature which is likely to produce memory effects rather than the excitation of biological oscillators. Therefore, at least 2 processes are at work in this single celled organism, where one is slow moving and one is much quicker. The oscillators would be tuned by the viscosity channels, that would maintain a behaviour until the channels themselves changed and this slower change is more structural. Figure 2 illustrates how the neuron and network transpose from ensemble to type, and the amoeba may indicate a precursor to neural synapses, for example.

## 5. Neural Binding

This is an important question from both the psychological and the mechanical aspects of the human brain. It asks why the brain does not confuse concepts like ‘red square’ and ‘blue circle’ unless these are fully defined by brain patterns first. Why is ‘red’ and ‘blue’ not confused, for example. The problem is that it would not be possible to store every memory instance combination in the brain and so (dynamic) linking of concepts is required. The paper [15] includes the idea of consciousness and how the brain is able to be coherent. Some models may include temporal logic or predicate calculus rules to explain how variables can bind with each other. Quantum mechanics is another plausible mechanism for merging patterns [25]. The paper [14] is quite interesting, where they describe a framework called the Specialized Neural Regions for Global Efficiency (SNRGE) framework. The paper describes that ‘the specializations associated with different brain areas represent computational trade-offs that are inherent in the neurobiological implementation of cognitive processes. That is, the trade-offs are a direct consequence of what computational processes can be easily implemented in the underlying biology’. The specializations of the paper correspond anatomically to the hippocampus (HC), the prefrontal cortex (PFC), and all of neocortex that is posterior to prefrontal cortex (posterior cortex, PC). Essentially, prefrontal cortex and the hippocampus appear to serve as memory areas that dynamically and interactively support the computation that is being performed by posterior brain areas. They argue against temporal synchrony, because of the ‘red circle blue square’ question and prefer to argue for coarse-coded distributed representations (CCDR) [26] instead. With CCDR, the concepts themselves can remain separate and it is not necessary to declare every binding instance explicitly, but it can be obtained from a local overlay coding scheme. The author has argued that patterns can be aggregated to some extent ([4] and earlier papers), when manipulation of them can then be done with much fewer neural connections over the aggregated representations. He has also argued that simply 2 layers with the same node representations can produce the required circuits. However, to realise these two concepts, still requires linked formations that either contain red and square, or blue and circle and so CCDR looks like a neat solution to this. However, it might be a question of whether the links are permanent or created ‘on the fly’. There is also the problem with imagination that can create new images. If the ensemble structure does not exist, then it would have to be constructed dynamically.

**Figure 3:**
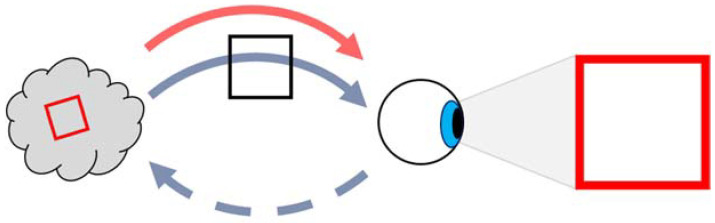
The brain sends two signals to the eye to construct an image and receives the feedback of this.

The author has also argued, or asked, why the senses are not part of the human conscious. Recent science however, is beginning to suggest that the whole nervous system is the conscious. We have eyes, ears, voice box, and so on, which we use as external mechanisms to the brain function and the paper [27] argues that when the brain thinks, it sends signals back to these organs and senses, and that they are essential to realise what the brain is thinking. If we consider the ‘red square, blue circle’ problem again, then one problem with current philosophy may be that we assume the pattern formations are translated only by the brain. One problem with that is the fact that the conscious would have to see every pattern and pattern part as the same. It would then require additional capability to try to differentiate. The ‘red’ concept has the same makeup as the ‘square’ concept to the brain conscious, for example. If it is possible to introduce different functions to the problem, then a solution may be easier to find and for the author, this would mean feedback to the external sensory organs. Considering the eye and for the sake of argument, let it produce only image shapes and colours. What if one signal could request the eye to produce an imprint of a shape on it and then a second separate signal requests that the eye gives it a particular colour. If this was possible, then the two signals would not necessarily have to be linked first, where that requirement has changed over to one about sending different function requests to the eye. This example is illustrated in Figure 3 and would make the neural-binding problem much easier, because the orthogonal function requests do not require all of the combinations that the more holistic conscious might require. Part of the binding has been moved to the eye itself. It may be the case that this is only for long-term memory, where a holistic memory store of recent images would still operate. Additionally, with this setup, the functional signals do not need to be fully linked patterns, but can be single links, for example, while the concept patterns can still be fully linked.

## 6. Cognitive Model

The author has developed a cognitive model over several years. The original design [24] had an architecture of 3 levels of increasing complexity, but also a global ontology that any of the 3 levels could use. The idea of an underlying global representation raises some interesting ideas. The author’s background is in computer science, distributed systems and also the Internet, where the SUMO ontology [28] and others, have been previously suggested. SUMO (Suggested Upper Merged Ontology) has been created to be a common language for the Internet. It is more expressive than object or semantic recognition, but not as much as natural language. Base concepts include ‘object’ and ‘process’, for example, but being an ontology, it includes relations between the different ontology entities. The author has worked on a cognitive model that is now at an early stage of development, with an even simpler ontology at its base. It is not even an ontology, but levels of symbolic node clusters, where a lower-level contains more frequently occurring symbols or concepts. The clusters are not linked together, but they offer some kind of global ordering over the stored symbolic representations. One example may be the 3 short stories—‘fox and crow’, ‘fox and stork’ and ‘fox and grape’. If a basic word count is done on each story, then for the ‘fox and crow’ story, the crow word occurs more frequently and so if using the concept trees counting rule (any child node in a tree cannot have a larger frequency count than its parent), it would be placed as the root tree node. In a global sense however, the fox word is more common, because it occurs in all 3 stories. Therefore, the global ontology would in fact re-order the local ‘fox and crow’ instance, to place ‘fox’ at the root node and then ‘crow’ one level above that. For this architecture therefore, the local story instance provides what concepts are available, but they are then re-ordered by the global ensemble. It is the same idea as the natural ordering for concept trees, described in section 6.4 of the paper [29]. With that, a road would always be placed below a car, for example, because a car would run on the road. With the cognitive model implementation therefore, there is the global ensemble of concepts at the base as the memory structure. Each of the 3 cognitive levels—pattern optimisation, tree-pattern aggregation and more complex concepts—also write a simplified view of their structures to the memory database. Then, when any level wants to read from memory, it uses the global database to retrieve whatever memory type it requires. The global memory structure therefore has different levels of representation that reference the ensemble clusters. It is also a common view of the information in the system, where any module can read and understand what a memory node is, because the more complex functionality that may be specific to any module is missing.

### 6.1. Natural Structure

This is also in the context of the cognitive model. Considering the author’s earlier work, the ReN (Refined Neuron) [1] has not been considered recently. The original idea was to make the signal more analogue, but it has become clear with biological modelling of the neuron that it can produce variable signals by itself. What may not be clear however, is if this is in discrete signal bands or a continuous signal. Discrete bands would match better with a type-based approach, when the ReN may still be useful. The other idea was that it is caused by repulsion of the signal down the input channels, which would be the axons and that would encourage new outlet paths to form. A third idea of balance is implicit in any energy system, even before the biological world.

The idea of frames (Minsky, [1]) is still interesting for the cognitive model. If it was used as part of the memory structure, then it would produce distinct units, including terminal states. The author has suggested a frequency grid classifier [3], which is entropy-based, reducing a global error, but one that is event or experience-based. It is a self-organising method that clusters elements with other elements that they are most often associated with. It was also suggested that it would be the base of a ‘unit of work’ that is a unit of ensemble-hierarchy structure. The ensemble-hierarchy structure [3,4] was originally intended to produce a more combined and analogue signal, but from Newtonian mechanics rather than a Quantum effect. The hierarchy would repeat the ensemble nodes, but with an additional tree structure and then resonating between similar node sets in both parts would produce ‘notes’ that would be recognised by the conscious. This is really very similar to the relationship between astrocytes and neurons [11], for example. Resonating is not part of the current research, but the ensemble-hierarchy is still an important structure. The tree nodes might become abstracted representations of ensemble patterns instead, where the structure adds meaning to what is otherwise a flat or nested matrix. In this sense, the ensemble-hierarchy would probably be expected, rather than being novel and it also fits better with a tokenised memory.

It may be interesting to note that Hill et al. [9] discovered that the connectome of cortical microcircuitry is largely formed from the nonspecific alignment of neuron morphologies, rather than pairwise chemical signals. This means that structure is preferred, whereas the signal is more dynamic. They also discovered that, although the specific positions of synapses are random, the restrictions caused by structure and neuron type, serve to ensure a robust and invariant set of distributed inputs and outputs between pattern populations. This would be grounded in biophysics. If the neuron synchronises more with static structure, then this will help it to maintain both form and lifespan [1], which is again a favourable conclusion for the ensemble-hierarchy relationship. The paper [2] then showed that it is more economic energy-wise, to produce a new neuron half-way between other neurons. This would also help to keep the path lengths regular, which again helps the neurons to synchronize their firing. If a particular region became active and started producing new neurons, that would change the path lengths, but the lengths would still remain quite regular and therefore recognisable as a type. Therefore, a lot of intelligence can be derived automatically from the structure, before even considering the neural functions. The author also wonders if distance between neurons is part of the pattern type itself. If, for example, the same chemical travels round a network of closely packed neurons or sparsely packed neurons, would that represent a different type to the brain? It would certainly change the relative strength of the signal, but also firing rates and timing.

### 6.2. Natural Function

The world therefore appears to be typed, even at the lowest level. For an amoeba, it may be a single type, whereas for humans, it is ensembles of types, but it is necessary to be able to discriminate over this. Order is another low-level process, not a high-level one. Not only order, but also regularity, where there is a sense of learning from repetition. Worms for example, have a behavioural order and ants make use of both of these functions, where collectively, they appear to exhibit intelligence. Feedback is also essential, where even at the cell level, there may be a necessity to express oneself. Thus far, we have energy optimisation, object or type recognition, spatial awareness, feedback, timing and ordering. Then, intelligence appears to be the evaluation of these lower-level processes, where there are obviously different levels of intelligence. The bee, for example, has a more developed brain with modules that are also recognisable in the human brain [22], but its reasoning process must surely only be at a logical level.

## 7. Natural Language Development

In the human brain, there are cells other than neurons, such as the more-simple glial and interneuron cells. More recently the perineuronal network [13] has received a lot of attention and may be exactly the memory structure that the cognitive model will now use. If the memory structure is sightly separate therefore, this can lead to at least two different information flows, for either memory or function. If the Perineuronal network is made of the glial cells—astrocytes and deodendrocytes, for example, then astrocytes are also known to produce energy for neurons and so successfully syncing with the memory structure would also provide an energy supply. The author’s own cognitive model implements a similar type of architecture, described in Section 6. Through implementing the cognitive model, it was interesting to note some separation between the global representation and the original sources, and also a little bit of autonomy for the global representation. Tokenized text, for example, might be stored largely as nouns and verbs, without all of the natural language. The architecture also works with images. The author is using a new idea called Image Parts [30], which scans an image and splits it up into parts, but is currently only useful for object recognition. The parts can then be stored in the global ensemble database and re-used. To re-construct an image representation, one part may be north of another part, for example. The algorithm is not very accurate, achieving only 80% accuracy, where neural networks would achieve closer to 100% accuracy, but it is also explainable. When other modules want to interface with the image, they can make use of the same ensemble parts, structured by an abstract tree representation.

The author postulates that this is like the brain architecture itself making use of a common language, to allow the different modules to interact with each other. The homogeneous input is converted over to a different tokenised representation, that is then used to describe the input to any other part of the system. If that process is internal to the brain itself, then it may be a reason why humans have developed their natural language, in order to try to express this internal structure. Nouns and verbs are the basis of the real world as well, for example and the paper [31] concludes that: ‘The available studies on the neural basis of normal language development suggest that the brain systems underlying language processing are in place already in early development’. This suggests that the structure for natural language is in place from a very early age. The paper [32] states that deep learning algorithms can produce, at least, coarse brain-like representations, suggesting that they process words and sentences in a human-like way. Word vectors may be superior to tree linking, but it is still a distributed and tokenised AI algorithm that can be mapped to brain regions. Problems have also been found with the design. Bees are also thought to communicate using a symbolic language that results in their waggle dance. Like the amoeba then, did they reason that they should communicate this, or is it a reflection of their internal structure? Maybe it is just an evolutionary quirk.

## 8. Conclusions

This paper gives a narrative that outlines structural components of simpler organisms that may have helped the human brain to evolve. More than that, the structures are so basic, they can be included in a computer model for Artificial Intelligence and are consistent with the author’s own cognitive model. The design may not be 100% accurate, but there appears to be a consistency about it and some biological and mathematical evidence can help to validate the theories. An early idea about scheduling through nesting may be seen in action in worms, for example, but in a simpler form. Then, one idea may be that intelligence can be realised automatically by converting from ensemble input to type-based output. This would occur automatically in the neuron network, where the realisation of types will produce some understanding and therefore intelligence. Amoebas are able to learn single types. The stigmergic processes of termites or ants, for example, have become interesting to explaining the neural structures for several reasons. Firstly, it is suggested that the neural microcircuitry is constructed primarily from the alignment of morphology or structure, rather than signal type and this includes synapse alignment and preparation. Although, the chemical signal will still change the type emitted by the cell. Secondly, the relationship between neurons and the substrate of glial cells, for example, also suggests stigmergic processes.

It would be interesting if there is an underlying global memory structure to the brain, which is this perineuronal substrate and if it can abstract and even re-structure input signals. The uniformity of the substrate would allow it to communicate this to other modules and a computer model would be able to simulate it to some level. When modelling the biological structure, images may be stored as whole representations in the short-term memory, but when they are moved into long-term memory, they become tokenized and abstracted. One final idea is that the neural binding problem is constrained by current thinking about a holistic conscious and if it can be made more orthogonal and receive help from other organs, the problem will become much easier to solve.

Most interesting then may be the idea that a cell or organism evolves, not only to survive, but also by expressing itself, where the expression is a result of its own internal structures and processes. In this respect, the memory substrate would be a precursor to our own natural language and this might also be seen in bees. The structural transformation from input to tokenized ensemble results in a communication process that is akin to a common language. The higher cognitive processes, if you like, have built themselves on the lower-level structures and processes.

## Figures and Tables

**Table 1:** Relative Pattern Strengths after Firing Sequences.

Neurons	*t* = 3	*t* = 4	*t* = 5
1	7.5	5.0	0.0
2	7.5	5.0	0.0
6	7.5	7.5	5.0
7	7.5	7.5	5.0
11	5.0	7.5	7.5
12	5.0	7.5	7.5
16	0.0	5.0	7.5
17	0.0	5.0	7.5
21	0.0	0.0	5.0
22	0.0	0.0	5.0

## Data Availability

Not applicable.
